# GlAIcomics: a deep neural network classifier for spectroscopy-augmented mass spectrometric glycans data

**DOI:** 10.3762/bjoc.19.134

**Published:** 2023-12-05

**Authors:** Thomas Barillot, Baptiste Schindler, Baptiste Moge, Elisa Fadda, Franck Lépine, Isabelle Compagnon

**Affiliations:** 1 Univ Claude Bernard Lyon 1, CNRS, Institut Lumière Matière, F-69622 Villeurbanne, France; 2 Department of Chemistry and Hamilton Institute, Maynooth University, Maynooth W23 F2H6, Ireland

**Keywords:** Bayesian neural network, deep learning, glycomics, IR, spectroscopy

## Abstract

Carbohydrate sequencing is a formidable task identified as a strategic goal in modern biochemistry. It relies on identifying a large number of isomers and their connectivity with high accuracy. Recently, gas phase vibrational laser spectroscopy combined with mass spectrometry tools have been proposed as a very promising sequencing approach. However, its use as a generic analytical tool relies on the development of recognition techniques that can analyse complex vibrational fingerprints for a large number of monomers. In this study, we used a Bayesian deep neural network model to automatically identify and classify vibrational fingerprints of several monosaccharides. We report high performances of the obtained trained algorithm (GlAIcomics), that can be used to discriminate contamination and identify a molecule with a high degree of confidence. It opens the possibility to use artificial intelligence in combination with spectroscopy-augmented mass spectrometry for carbohydrates sequencing and glycomics applications.

## Introduction

DNA and protein sequencing technologies that aim at determining the structure of a biopolymer have been established decades ago and are commonly used in a routine and automated manner. However, the development of such technology for the sequencing of the third class of biological polymer – glycans, also known as carbohydrates, saccharides, or "sugars" – lags far behind. This lack of dedicated analytical tools (glycomics) is clearly identified as a critical bottleneck, impeding the full development of glycosciences despite their relevance for various strategic fields such as pharmaceutical and food industry; bio-based materials and renewable energy, and their considerable potential impact for the society in regard to the United Nations sustainable development goal [1].

The major roadblock to carbohydrate sequencing is intrinsically due to their unique molecular properties, among biopolymers. In contrast with proteins and DNA, which are linear polymers made of a limited number of building blocks with distinct molecular structures, carbohydrates feature hundreds of building blocks – many of them coming in groups of closely related isomers with ambiguous molecular structures – and they form complex, branched arrangements due to the versatility of the glycosidic bond (position and anomericity). In this context, designing generic carbohydrate sequencing methods is both a major scientific challenge and a strategic priority [2–3].

Few years ago we proposed an original solution by bringing together the best of both sides of the analytical chemistry world: Spectroscopy and mass spectrometry (MS). In short, our technology is based on a mass spectrometric analysis – which is particularly powerful for the analysis of complex biological samples but does not readily elucidate isomers which have the same molecular mass – augmented with a infrared laser-based spectroscopic dimension (MS–IR), thus providing valuable additional isomer resolution [4].

We demonstrated that this multidimensional MS–IR molecular fingerprint is unique to each carbohydrate building block and can be used to resolve their full sequence, including their monosaccharide content and the detail of their linkages (position and anomericity). Based on this basic principle, the identification of an unknown carbohydrate proceeds as follows: the polymer is fragmented in monomers, yet maintaining information on the initial structure and the spectroscopic fingerprint (frequency and intensity of the vibrational modes) of each monosaccharide unit is measured, and subsequently identified by comparison with a library of reference spectra of synthetic monosaccharide standards. In the early days of MS–IR spectroscopy, ca. one hour was necessary to record the IR fingerprint of a single molecule and the identification was made by visual inspection, which was shortly automated by introducing a score derived from the convolution between the spectrum of the analyte of interest and the library of reference spectra. Despite the advantage of being automated, this later approach remains biased: for each molecular species, a single spectrum is arbitrarily chosen by the operator and serves as reference for all future analyses.

The latest MS–IR developments brought the data collection down to few seconds [5]. This is a considerable step towards high throughput carbohydrate analysis, which must be accompanied by fast data analysis, thus excluding manual interpretation. Besides, in the prospective of deploying the technology beyond the molecular spectroscopy community, it is essential to develop an automated, reliable, and robust strategy for the analysis of the spectroscopic data. Machine learning methods appear to be appealing candidates to address this challenge. They have been used for mass spectrometry data analysis since the 2000’s [6] and the idea of using them on vibrational spectra goes back to the early 90’s [7]. Support vector machines (SVM) and decision tree ensemble methods were benchmarked on infrared spectra for cancer classification [8] and many research groups focused their efforts on using machine learning for simulating molecular structures; generating vibrational spectra; and classifying chemical groups based on vibrational features [9–10]. In a recent publication, the random forest approach was proposed to identify the presence of structural features in oligosaccharides based on their gas-phase IR spectra [11]. To the best of our knowledge, machine learning classification studies have not been reported to identify saccharides using MS–IR carbohydrate analysis.

Here, we report a study of a probabilistic deep neural network (Bayesian deep neural networks [12]) to support automated monosaccharide recognition for carbohydrate sequencing. We obtained a highly performing algorithm that we called "GlAIcomics", specifically trained on carbohydrates.

## Methodology

### Data production

**Figure 1:**
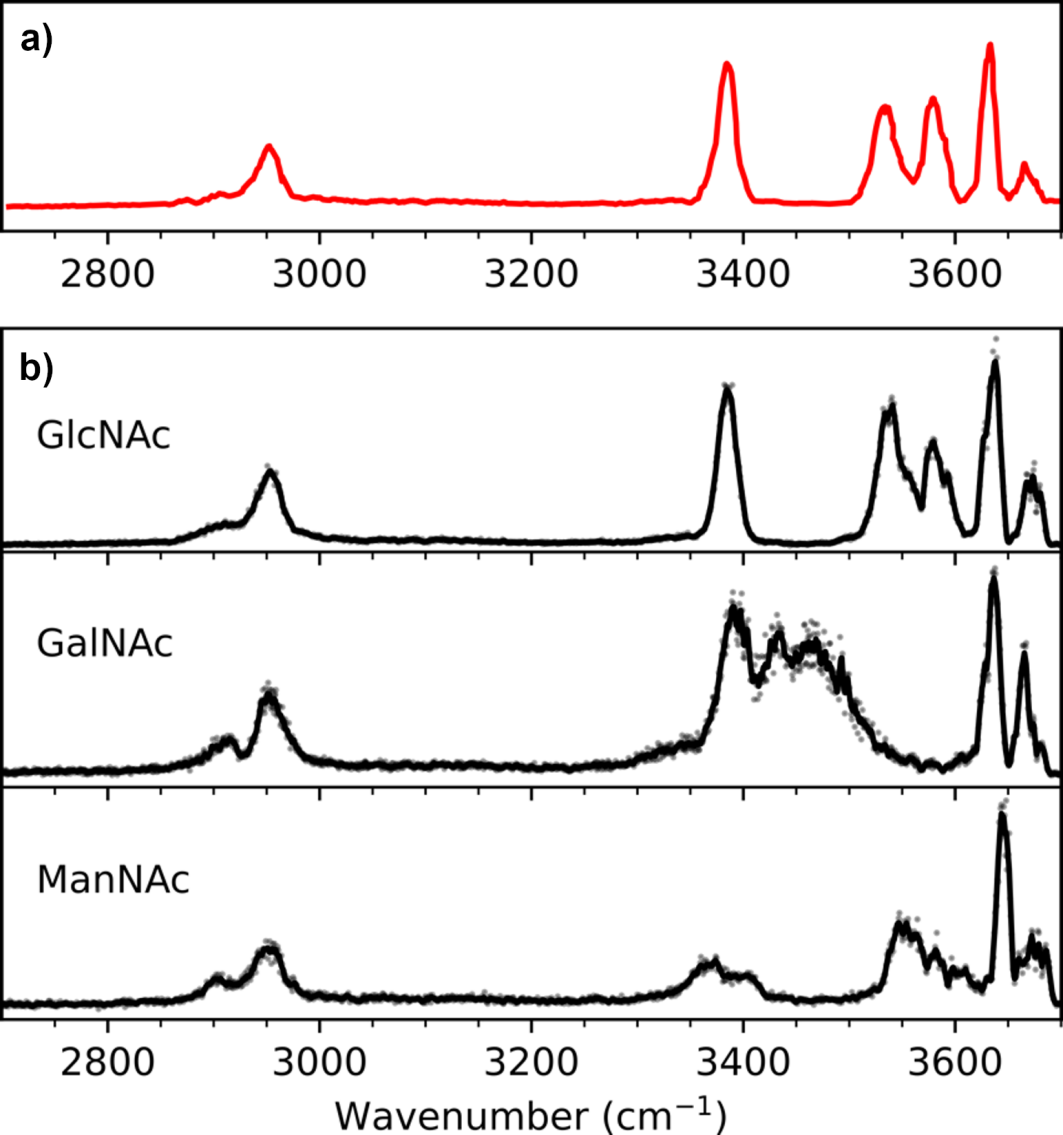
(a) Fingerprint of an unknown monosaccharide. (b) Labelled reference spectra of monosaccharide standards.

Our carbohydrate analysis approach is based on the IRMPD spectroscopic scheme (infrared multiple photon dissociation), which is the combination of mass spectrometry and IR spectroscopy. IRMPD is an action spectroscopy method that allows recording IR absorption spectra of isolated gas-phase ions, based on the measurement of the wavelength-dependent laser-induced fragmentation yield. When the frequency of the laser is resonant with a vibrational mode of the molecule, the molecule absorbs the radiation and accumulates internal energy until fragmentation [13]. In previous works we have demonstrated that the monosaccharides or oligosaccharides resulting from the fragmentation of a larger precursor possess a very specific IR fingerprint in the 2–4 microns spectral range, that is highly valuable to resolve all types of isomers [4]. Typical experimental IR fingerprint data are shown in Figure 1: they feature the intensities of the vibrational resonances as a function of their frequency in the mid-IR range. After measuring its mass and its IR fingerprint, an unknown analyte (Figure 1a) is readily identified as "GlcNAc" (for *N*-acetylglucosamine) by comparison with the reference IR spectra of several candidates of identical mass (Figure 1b, featuring three stereoisomers of C_8_H_15_NO_6_). With the rapid development of our approach, such method now reached a high data output since a single IR fingerprint can be obtained in few seconds. The fast and automatic identification and classification of the data becomes compulsory, which motivates the present study.

**Figure 2:**
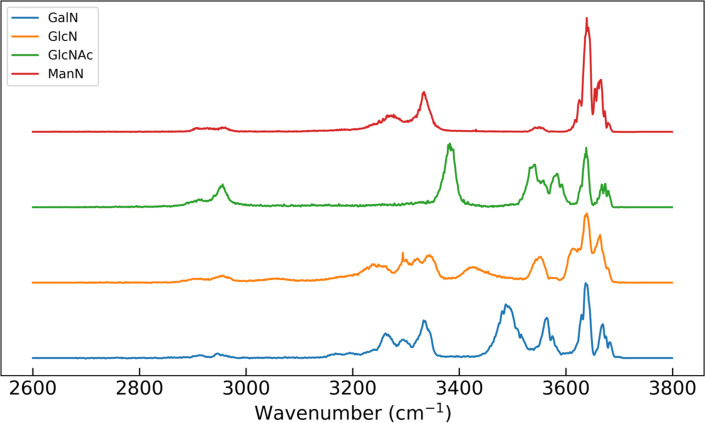
Typical experimental MS–IR spectra of the four categories of monosaccharides included in the first dataset. Blue: GalN; orange: GlcN; green: GlcNAc; red: ManN.

For this study, a first set of 33 labelled experimental spectra obtained as described previously [4] were collected for training and validation of the model. The standard instrumental conditions for recording MS–IR data consist in a laser-enabled mass spectrometer equipped with a 3D ion trap mass analyzer. The following monosaccharides were analyzed: three stereoisomers of hexosamine of chemical formula C_6_H_13_NO_5_, namely glucosamine (GlcN), galactosamine (GalN), mannosamine (ManN); and *N*-acetyl glucosamine (GlcNAc, chemical formula C_8_H_15_NO_6_). One typical spectrum of each of the four monomers is shown in Figure 2. Note that both α and β-anomers coexist in the experimental conditions.

**Figure 3:**
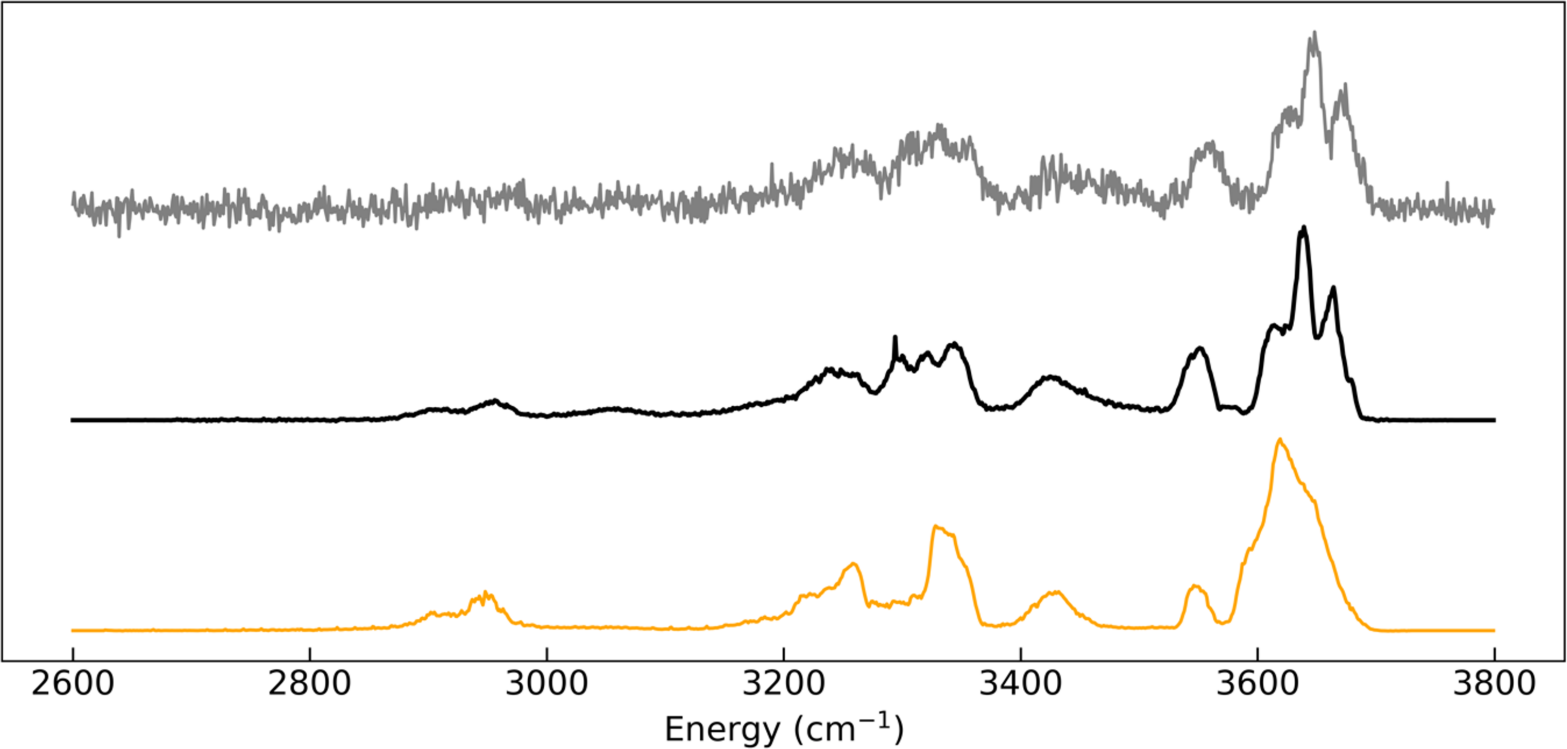
Synthetic IRMPD spectrum (grey trace) generated on the basis of a high resolution endogeneous experimental spectrum of GlcN (black trace) from dataset 1 using additional white noise: 10%; linear signal amplitude modulation: 5%; downsampling coefficient: 2; wavenumber shift: +9 cm^−1^. The orange trace corresponds to a low-resolution exogeneous GlcN spectrum from dataset 2.

The second set of experimental MS–IR spectra was acquired using different instrumental conditions on a different experimental set-up: it consists of the coupling of an alternative design of mass spectrometer (equipped with a 2D ion-trap mass analyzer) with a higher repetition rate laser and a larger spectral bandwidth [5]. New GlcN spectra were acquired in these conditions. One of them is shown in Figure 3 (orange trace) for comparison with an experimental spectrum of GlcN acquired in standard conditions. Due to the larger spectral bandwidth, the spectrum from set 2 looks significantly different: the peaks are broader and less resolved than in the spectrum from set 1. This set is referred to as exogenous and was not use for training: it is used to illustrate the robustness of the method across significantly variable experimental conditions and instrumental performance.

The third set of experimental IRMPD spectra was acquired in standard conditions and includes 5 new spectra from the monomers GlcN, GalN, and ManN as in sets 1 and 2; as well as 7 spectra from species that do not belong in the training set categories (out of distribution, OOD), including disaccharides, a sulfated monosaccharide, and paracetamol. The outlying molecules represent potential "pollutions" in the analysis. This set of data is referred to as endogenous as it was measured on the same apparatus as the training set.

For efficient training of the algorithms, all three experimental datasets were augmented by producing synthetic variants. These synthetic spectra were generated by modulating the experimental ones with the following relevant sources of experimental fluctuations:

- The signal to noise ratio may vary from one measurement to another as it can emerge from a low amount of molecules. This was simulated by adding a Gaussian white noise with a randomly distributed standard deviation between 0 and 5% of the peak signal.
- The overall intensity of the laser can fluctuate from day to day or thorough the entire spectral range, which results in modulated peaks amplitudes. This was simulated as a linear variation of the signal amplitude across the spectral range. The variation was contained in a uniform distribution bounded by ±10%.
- Spectra can be recorded at increased speed for rapid analytical diagnostics, which traduces into a change in binning. To take this into account, data were binned with downgraded resolution then re-binned with 1 cm^−1^ step. The down sampling factor was randomly picked in a range from 1 to 5.
- Small variations of the calibration of the laser wavenumber may occur from day to day, leading to a shift of few wavenumbers of the vibrational spectrum. This was simulated with a maximum random shift per spectrum of ±10 cm^−1^.

Finally, the synthetic spectra were normalized by z-score and interpolated over 1200 bins in the 2600–3800 cm^−1^ spectral range (1 cm^−1^ step) as input vector for the neural network. An example of a synthetic spectrum generated from an experimental spectrum is shown in Figure 3.

A total of 8000 synthetic spectra were randomly produced (2000 for each monomer category) out of the experimental spectra of set 1. They were shuffled to avoid training batches composed of a unique category of molecules. Finally, 70% of them were used for training of the models, and 30% were used for validation. The composition of the datasets used for training, validation and tests is summarized in Table 1.

**Table 1:** Composition of the three datasets.

	Dataset 1	Dataset 2	Dataset 3

	training : 70% validation : 30%	classification tests	discrimination tests

categories	4	1	10
acquisition	standard	low res.	standard
exp. MS–IR spectra	33	4	12
augmented set	8000	8000	1300

### Model architecture

In this study we opted for a fully connected feed-forward network based on the multi-layer perceptron architecture [14] with probabilistic approach (Bayesian deep neural network, DNN), which allows quantifying the model uncertainty for the classification results. It is composed of 3 hidden layers of 300, 225, and 100 neurons, respectively, and ReLu (rectified linear unit) activation functions for each layer. Two dropout layers are interleaved after the first and second hidden layers with a dropout setting of 25% to avoid over-fitting issues. The training objective is a classification task between the 4 monomer categories with a cross-entropy loss function.

To account for the probabilistic nature of the deep neural network, we used the variational inference technique. Each deterministic weight parameter was replaced by normal distributions defined by a mean value µ and a standard deviation σ which were optimized using the Bayes-by-Backprop method [15]. We chose this method that constrains the weights posterior distribution to normal distributions instead of the more accurate Markov chain Monte-Carlo (MCMC) method for calculation efficiency. With this approach, a quantitative uncertainty of the model predictions can be achieved by inferring each spectrum category several times with the trained model.

## Results and Discussion

### Model classification accuracy

**Figure 4:**
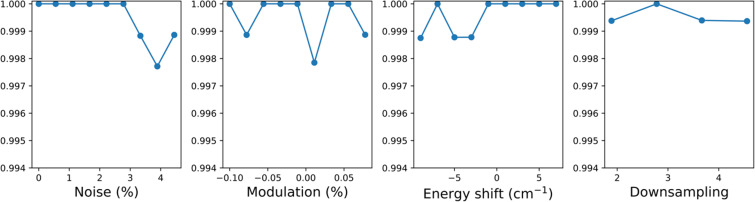
Model accuracy dependance with experimental conditions, represented by the dataset augmentation parameters.

Our GlAIcomics model shows a classification accuracy of 100% on the validation set and 99.98% on the test set (S.M : dataset 2 in Table 1). The 8000 synthetic spectra of set 2 were sorted by noise level, amplitude modulation, energy shift, and downsampling. The mean accuracy of the model as a function of these four parameters is shown in Figure 4. Note that all parameters have a uniform distribution over the 8000 samples and can be studied independently. The amplitude modulation and downsampling do not play a major role, with a maximum accuracy variation of 0.5%.

We demonstrated that the neural network is suitable for MS–IR classification in experimental conditions with variable resolution, noise or energy jitter.

The question remains on how to discriminate unknown molecules or to identify problematic spectra, such as the few misclassification events in the discussion above. In order to address these points, we further assessed the precision of the model and discussed its epistemic uncertainty in the next section.

### Model precision and uncertainty

In the context of analytical chemistry where the fraction of "known molecules" (that is, previously referenced in databases) is expected to be significant compared to unknown ones, it is important to make sure that the model is discriminative and we want to maximize the precision of the model at this task. Indeed, the large amount of positive results would make it difficult to identify false positives. However, a small number of negative results is expected, which makes it doable to assess them systematically. False negative could be identified manually, labelled correctly, and injected back to improve the model.

The third dataset was used to evaluate the model discriminative power. It consists of 1300 spectra produced by augmentation of 12 original experimental spectra that were acquired on the standard instrumental setup and were never used by the models during the training and validation phases. This set contains 3 of the 4 known monosaccharides: ManN, GlcN, and GalN as well as 8 other molecules. For benchmarking purposes, all spectra were annotated with true labels.

**Figure 5:**
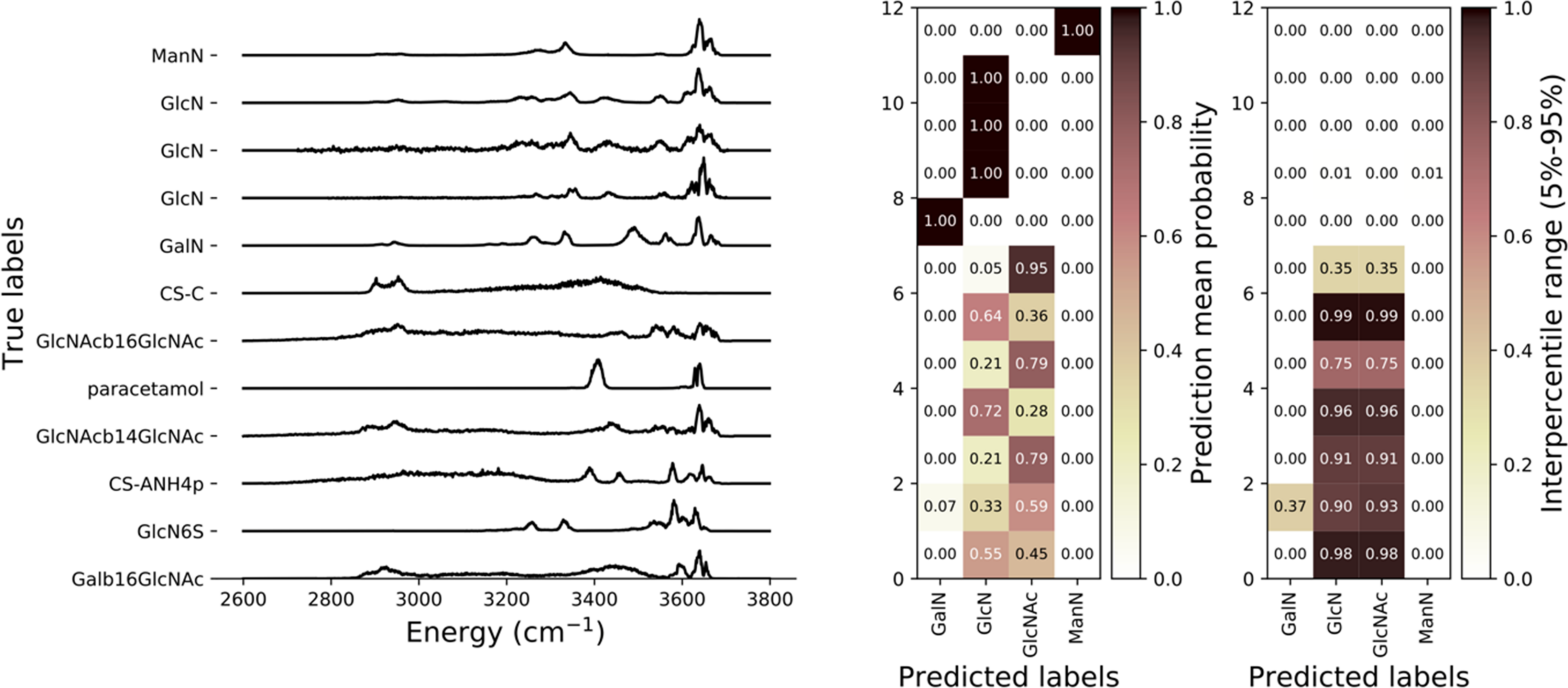
DNN Prediction results for third endogenous dataset (5 hexosamine samples and 7 other molecules). The middle map shows the mean prediction probabilities for each category and the right hand side map shows the 5% to 95% interpercentile range for the prediction probability distributions of each category.

By running the model inference for one spectrum multiple times we can measure the variability of its prediction probability for each category. If the model gives consistently a high probability for one category after each inference, then its uncertainty is low, and the spectrum likely belongs to the said category of molecules. On the other hand, if the model predicts a category with highly variable probability, then the uncertainty is high, and the spectrum likely does not belong to any of the classification categories. We ran model inference 200 times on each sample and obtained the mean prediction probability for every category as its variability represented by the interpercentile range 5 to 95%. The results are shown in Figure 5. As an example: the spectrum of CS-C is predicted as GlcNAc with 95% probability in average but for 10 inferences out of 200 (the lowest 5% percentile) the prediction probability is below 60%. In this example, by thresholding on the interpercentile range below 0.35 for the most likely prediction of each spectrum one can obtain a precision of 100%.

Most known molecules are assigned to the right category with a very sharp probability distribution that can be used as the prediction distribution under the null hypothesis that the model is reliable. For most of the "unknown" molecules the model prediction oscillates between two categories, but the probability distributions are extremely broad which means that the neural network uncertainty is important, and the corresponding results should be considered as unclassified and put aside for manual evaluation.

Finally, the performance of the GlAIcomics deep neural network model was compared with two different off-the-shelf techniques based on decision trees: Random forest (RF), an XGBoost (XGB). The evaluation methods are detailed in Supporting Information File 1. The classification accuracy for the validation subset (30% of set 1) is 100%, 99.95% and 100% for RF, XGBoost and GlAIcomics, respectively. For the test set (dataset 2), the accuracy is 99.91%, 99.61%, and 99.98%, respectively. When the accuracy of the prediction is further investigated as a function of the data augmentation parameters used to model experimental fluctuations, an advantage is found for GlAIcomics and RF over XGBoost. Lastly, the three methods were compared for the discrimination of molecules outside of the known categorie. GlAIcomics appears to discriminate samples more efficiently than the two other methods with true and false positive rates above 80% (70% and 50% for RF and XGBoost, respectively).

## Conclusion

We have evaluated the performances of a Bayesian deep neural network for automatic analysis and classification tasks on glycans MS–IR fingerprints. It showed robust prediction accuracies on an exogeneous dataset. We observed that it is capable to generalize as it could categorize more noisy and distorted spectra. We then benchmarked its discrimination capabilities with a mixture of hexosamines and other molecular spectra: the Bayesian neural network architecture offers an access to the model reliability (through its epistemic error) when it comes to classify the spectra and could be used to discriminate outlying molecules or experimental issues when run on new data samples. Therefore, we conclude that a relatively small Bayesian deep neural network is a suitable solution for analysis and classification of saccharides in the context of MS–IR based carbohydrate sequencing. It can be easily integrated in an experimental data pipeline between the experiment raw spectra recording and the sequencing algorithm. Rejected spectra would be manually reviewed and fed back to the model as new training samples which in turn would reduce the epistemic error. It will therefore speed up the construction of glycans spectroscopic fingerprints database. In MS–IR experiments, the IR data as well as the mass of the molecule are simultaneously acquired, therefore the mass could readily be used as a prefilter. More generally, all experimental data obtained in a glycomics workflow – such as MS/MS; HPLC; ion mobility; … – could ultimately be included in the algorithm for an optimal coverage of complex carbohydrates.

## Supporting Information

File 1 Evaluation of the deep neural network model against two different techniques based on decision trees: Random forest (RF) and XGBoost (XGB).
