# Supplementary material for: GlAIcomics: a deep neural network classifier for spectroscopy-augmented mass spectrometric glycans data
Source: Beilstein J Org Chem. 2023 Dec 5;19:1825–31. doi: 10.3762/bjoc.19.134 (PMC10714470; doi:10.3762/bjoc.19.134)
Supplement: File 1 — Evaluation of the deep neural network model against two different techniques based on decision trees: Random forest (RF) and XGBoost (XGB). [file Beilstein_J_Org_Chem-19-1825-s001.pdf]

BEILSTEIN JOURNAL OF ORGANIC CHEMISTRY

Supporting Information

for

GlAIcomics: a deep neural network classifier for spectroscopy-augmented mass spectrometric glycans data

Thomas Barillot, Baptiste Schindler, Baptiste Moge, Elisa Fadda, Franck Lépine and Isabelle Compagnon

Beilstein J. Org. Chem. **2023,** *19,* 1825–1831. doi:10.3762/bjoc.19.134

Evaluation of the deep neural network model against two different techniques based on decision trees: Random forest (RF) and XGBoost (XGB)

License and Terms: This is a supporting information file under the terms of the Creative Commons Attribution License (https://creativecommons.org/licenses/by/4.0). Please note that the reuse, redistribution and reproduction in particular requires that the author(s) and source are credited and that individual graphics may be subject to special legal provisions.
The license is subject to the *Beilstein Journal of Organic Chemistry* terms and conditions: (https://www.beilstein-journals.org/bjoc/terms)

Model architectures comparison

Classification accuracy

We evaluated the deep neural network model against two different techniques based on decision trees: a Random forest (RF)[1], an XGBoost (XGB) [2]. The Random forest (RF) and XGBoost (XGB) are both ”off-the-shelf” models from scikit-learn and xgboost libraries [2,3]. We performed a grid search with 5-fold cross-validation to select best hyperparameters for both models. RF was composed of 600 binary decision trees estimators with a maximum depth of 5, while XGB obtained its best performances for 100 estimators, a maximum depth of 4 and a learning rate of 0.2.

Table S1 presents the classification accuracy for the validation subset (30% of set 1) and set 2. RF and DNN successfully predict all the validation examples whereas XGB reaches 99.94% accuracy.

Table S1: Model accuracy for validation on the first dataset and testing on the exogenous dataset.

	RF	XGB	DNN
Validation (30% of dataset 1)	100 %	99.94%	100 %
Testing (dataset 2)	99.91%	99.61%	99.98%

The models performances and their ability to generalize were further tested on set 2, which is by construction fully uncorrelated with the initial training and validation sets: set 2 was produced from an independent set of experimental data, moreover, these data were acquired on a different instrumental setup. An accurate prediction of more than 99.5% was obtained for all three models on over 8000 augmented spectra.

We compared accuracies of all three methods as function of the data augmentation parameters we explored. It is illustrated in Figure 1 and shows an advantage of DNN and RF versus XGB. It is due to the fact that XGB only uses a limited amount of spectral bins to infer categories.

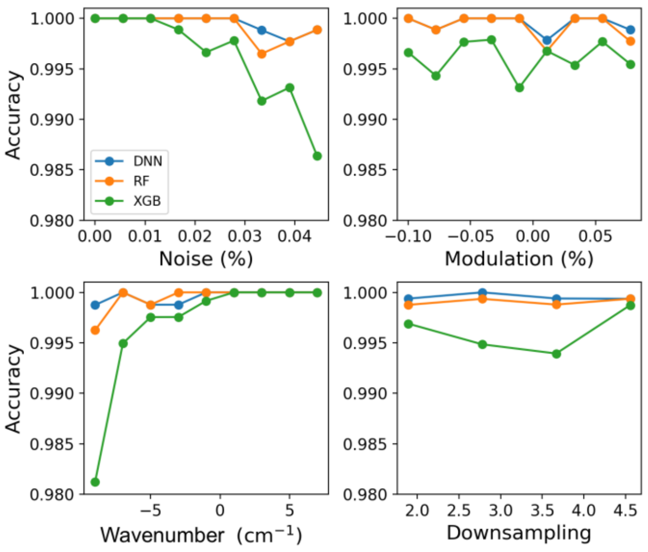

Figure S1: Model accuracies on test dataset as a function of data augmentation parameters.

Finally, we have compared the three methods over discriminating out of sample molecules from the 4 known categories. It can be represented as a binary problem. We have investigated which model performed best based on the mean predicted probability for the infered category. In the case of the two ensemble models (RF and XGB) it corresponds to the average probability over all estimators binary results. For the DNN, the model output probability distribution was computed for each category by running it 200 times on each sample and by extracting the mean value of the obtained distribution for the predicted category.

By varying a threshold value to discriminate ”good” spectra (properly predicted in one of the 4 known monosaccahride categories) from ”bad” ones (unknown spectra or wrongly predicted) on the basis of the mean prediction probability we could build a receiver operating characteristic (ROC) curve for each model (Figure S2) that shows the relation between the true and false positive rates (TPR and FPR) or sensitivity versus specificity.

The DNN appears to discriminate the samples way more efficiently than the two other methods: the area under the curve is maximized for this model and its TPR is above 80% for FPR = 0 when RF and XGB obtain ≈70% and ≈50%, respectively. Using the DNN therefore minimizes the amount of false negatives that would require manual audit and constitute a clear advantage over the other two methods in real experimental conditions.

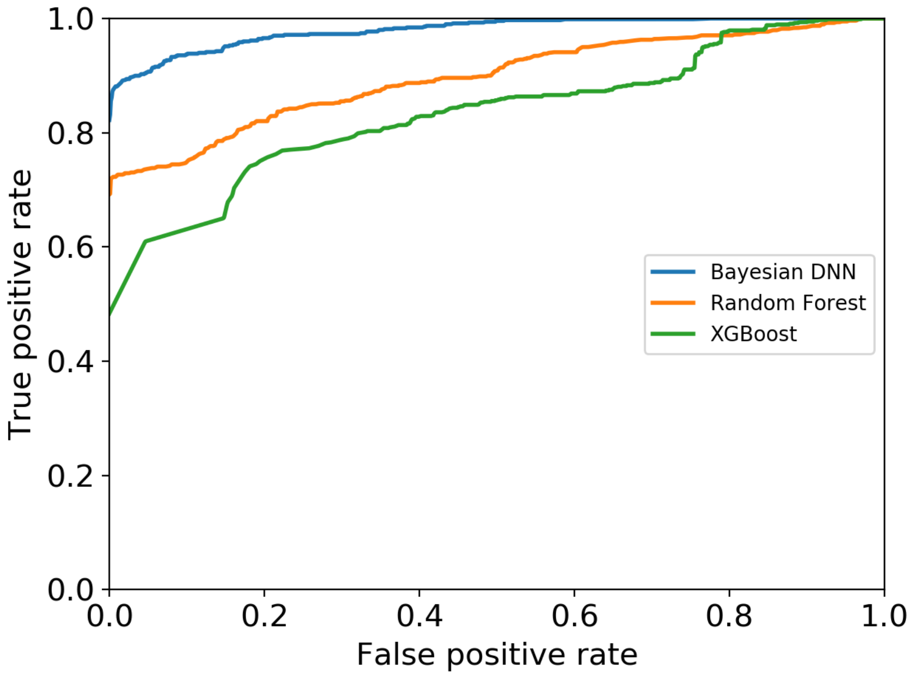

Figure S2: ROC curves for the DNN (blue), RF (yellow), and XGB (green).

References

[1] T. K. Ho, “Random decision forests,” in Proceedings of 3rd International Conference on Document Analysis and Recognition, IEEE Comput. Soc. Press.

[2] T. Chen and C. Guestrin, “XGBoost,” in Proceedings of the 22nd ACM SIGKDD International Conference on Knowledge Discovery and Data Mining, ACM, aug 2016.

[3] F. Pedregosa, G. Varoquaux, A. Gramfort, V. Michel, B. Thirion, O. Grisel, M. Blondel, P. Prettenhofer, R. Weiss, V. Dubourg, J. Vanderplas, A. Passos, D. Cournapeau, M. Brucher, M. Perrot, and E. Duchesnay, “Scikit-learn: Machine learning in Python,” Journal of Machine Learning Research, vol. 12, pp. 2825–2830, 2011.
